# Editorial on Antennas

**DOI:** 10.3390/s23249643

**Published:** 2023-12-06

**Authors:** Naser Ojaroudi Parchin, Chan Hwang See, Raed A. Abd-Alhameed

**Affiliations:** 1 School of Computing, Engineering and the Built Environment, Edinburgh Napier University, Edinburgh EH10 5DT, UK; c.see@napier.ac.uk; 2 Faculty of Engineering and Informatics, University of Bradford, Bradford BD7 1DP, UK; r.a.a.abd@bradford.ac.uk; 3 Department of Information and Communication Engineering, Basrah University College of Science and Technology, Basrah 61004, Iraq

## 1. Introduction

In the ever-evolving landscape of modern wireless communication systems, the escalating demand for seamless connectivity has propelled the imperative for avant garde, versatile, and high-performance antennas to unprecedented heights. These antennas, standing at the forefront of future communication systems, serve as linchpins that can potentially revolutionise the overall performance of communication networks [1]. The exponential surge in connectivity needs has instigated a notable surge in dedicated research endeavours, underscoring the paramount importance of advancing antenna technologies. The adaptability of these antennas, which is essential to meet the diverse and evolving requirements of wireless services, necessitates not only careful consideration but also a continuous stream of innovative approaches [2]. Navigating the dynamic terrain of wireless communication, antennas are required to embody a plethora of features to ensure efficacy in both current and future systems. These encompass expansive and multi-frequency coverage, compact form factors, meticulously defined radiation patterns, multi-mode operational capabilities, cost-effectiveness in fabrication, energy efficiency, streamlined integration and assembly processes, and conformity to ever-evolving standards [3]. The symbiotic integration of Multiple Input Multiple Output (MIMO) configurations and phased array arrangements, fortified by adaptive and smart antennas, stands as the linchpin for significantly augmenting system capacity, thereby adeptly meeting the burgeoning demands of the unfolding epoch of wireless networks [4].

As we delve into the vast expanse of antennas and their applications, we recognise their pivotal role in shaping the contemporary technological landscape. The advent of 5G and the looming prospect of 6G technologies underscore the escalating role of antennas in shaping the future of communication [5]. From applications in wearable devices and biomedical wireless communication to their indispensable role in the Internet of Things (IoT) and smart cities, antennas are catalysts propelling the world towards unprecedented connectivity and technological advancement. They are the silent architects of our interconnected future, laying the foundation for the seamless exchange of information in the realms of autonomous vehicles, intelligent infrastructure, and beyond [6]. In this continuous journey of antenna evolution, we are witnessing not just technological progress but a transformative force shaping how we connect, communicate, and innovate. Antennas, in their myriad forms, have become integral components in diverse applications. Their role extends beyond mere conduits of signals; they are the lifelines of global connectivity, enabling progress and ushering in an era of unparalleled interconnectedness [7]. The relentless pursuit of excellence in antenna design and technology is not merely a shared ethos but a driving force propelling the evolution of wireless technologies into uncharted territories. The future promises antennas that seamlessly operate across a broader range of frequencies, with increased energy efficiency and reduced interference. These antennas are not just technological marvels; they are the enablers of a future where connectivity knows no bounds, fostering innovation, collaboration, and a world united by the invisible threads of wireless communication [8].

The overarching objective of this Topic is to comprehensively cover all facets of antennas utilised in existing or future wireless communication systems. The aim is to spotlight recent advances, current trends, and potential future developments in antenna technologies. Within the expansive terrain of this Topic, our aim is to offer an inclusive panorama, meticulously detailing the most recent breakthroughs and pioneering methodologies in antennas. A compendium of thought-provoking contributions, totalling 46 papers, forms the backbone of this endeavour, collectively navigating the intricate landscape of antennas for emerging wireless communication systems. Each of these papers functions as a beacon, illuminating distinct facets and advancements within the realm of antennas. The myriad topics explored within this compendium contribute cohesively to the overarching objective of unravelling novel approaches, fostering a deeper understanding of the field and laying the groundwork for further exploration. In essence, this Topic aspires not only to document the present state of antenna technologies but also to propel the discourse forward, inspiring researchers to delve into the vast potential and uncharted territories that lie ahead.

## 2. Overview of Published Papers

The research from Zhao et al. (contribution 1) introduces an innovative approach to the pattern synthesis of linear array antennas. They propose the Nonlinear Chaotic Grey Wolf Optimization (NCGWO) algorithm, an enhancement of the Grey Wolf Optimization (GWO) algorithm, for optimal pattern synthesis. The NCGWO algorithm is demonstrated to outperform other intelligent algorithms in electromagnetic optimisation problems, providing superior performance in terms of global search capability and convergence rate. This work opens new avenues for efficient and effective optimisation techniques in antenna design, particularly for linear array antennas.

Moving on to contribution 2, the article by Wang et al. presents a significant development in W-band circularly polarised reflect-array antennas. The proposed antenna design achieves a remarkable 2 dB gain bandwidth of 27.6% and a 3 dB axial ratio bandwidth of 13.8%, making it highly suitable for wireless communication applications. The antenna’s performance, with a gain of 29.1 dBi and an aperture efficiency of 52.0%, positions it as a promising candidate for high-frequency communication systems. This contribution augments the ongoing efforts to broaden the applicability of reflect-array antennas in the W-band.

In contribution 3, Wang et al. address the demand for antennas with multiple operation bands and improved radiation gains. The authors propose a differentially fed, dual-wideband, dual-polarised patch antenna featuring a crossed dielectric resonator (CDR). The CDR design contributes to enhanced isolation levels and radiation gain, with the antenna exhibiting dual bands of 1.86–2.52 GHz and 3.26–3.72 GHz. The compact size of the antenna, coupled with its excellent performance characteristics, positions it as a promising candidate for 4G/5G wireless communication systems.

The paper by Abd Elrahman et al. (contribution 4) introduces two novel sector beam scanning approaches (BSAs) based on element position perturbations in the azimuth plane. These approaches offer a balance between scanning range and side lobe levels, providing a versatile solution for beam scanning applications. By combining element position perturbation with the single convolution/genetic algorithm technique, the proposed approach achieves a smaller scanning range with a relatively constant half power beamwidth and lower side lobe levels. This research contributes to the optimisation of beam scanning in linear antenna arrays.

The work by Yassin et al. (contribution 5) presents a flexible antenna designed for wideband biomedical wireless communication. The antenna, operating over the frequency range of 5–19 GHz, exhibits circular polarisation in the 5–6 GHz range and linear polarisation from 6 to 19 GHz. The proposed design, featuring an inverted G-shaped strip on a flexible substrate, achieves excellent performance metrics such as a 3 dB axial ratio bandwidth of 18%, impedance matching bandwidth of 117%, a maximum gain of 5.37 dBi, and high radiation efficiency. This flexible antenna design holds promise for diverse applications in off-body and on-body communication systems.

The research by Noor et al. (contribution 6) introduces a novel microstrip patch antenna design for sub-6 GHz and sub-7 GHz 5G wireless applications. Utilising slots and parasitic strips, the proposed antenna achieves enhanced gain and bandwidth, offering a wider bandwidth at both 3.45 GHz and 5.9 GHz compared to conventional designs. This innovation holds significant promise for high-gain compact patch antennas, contributing to the efficiency of 5G wireless communications in these crucial frequency bands.

In contribution 7, López-Álvarez et al. address the often-overlooked aspect of aperture efficiency in antenna design. Maximising aperture efficiency is demonstrated to reduce the required number of radiating elements, resulting in cost-effective antennas with increased directivity. The study introduces a mathematical expression for calculating aperture efficiency, emphasising its importance in antenna footprint patterns. This work provides valuable insights for the optimisation of antenna arrays, ensuring cost-effective solutions with enhanced performance.

The work by Mingle et al. (contribution 8) presents a multi-layer beam-scanning leaky wave antenna for remote vital sign monitoring at 60 GHz. This antenna, equipped with partially reflecting surfaces (PRS) and high-impedance surfaces (HISs), achieves a gain of 24 dBi and precise remote vital sign monitoring up to 4 m. The system’s ability to monitor vital signs in a dynamic environment, combined with its significant gain and scanning range, positions it as a promising technology for continuous health monitoring applications.

In contribution 9, Teodorani et al. propose a novel beam scanning architecture using a pair of planar metasurfaces for thin reconfigurable antennas. The design, employing on-plane varactor diodes, achieves beam scanning without the need for complex feeding networks. The demonstrated prototype in the X band offers an innovative approach to beam scanning, showcasing potential applications in satellite communications and 5G networks.

Han et al.’s work (contribution 10) presents a design method for low radar cross-section (RCS) array antennas based on characteristic mode cancellation (CMC). By introducing rectangular and cross-slots, the proposed microstrip elements achieve broadband dual-linear polarisation CMC, resulting in reduced monostatic RCS for dual-linear polarised waves. This design method offers a promising solution for low-RCS array antennas with improved bandwidth and radiation performance.

The paper by Abbas et al. (contribution 11) introduces a printed multiple-input multiple-output (MIMO) antenna for 5G millimetre-wave applications. The antenna, featuring a compact size and good MIMO diversity performance, operates in the ultra-wideband (UWB) range from 25 to 50 GHz. The orthogonal positioning of antenna elements enhances isolation, making it a suitable candidate for future 5G millimetre-wave applications.

Wang et al. (contribution 12) propose a simple yet effective beamwidth broadening technique based on an antipodal linearly tapered slot antenna (ALTSA). The design achieves a quasi-hemispherical radiation pattern without increasing the overall size and complexity. With only two rows of subwavelength metallic elements, the ALTSA presents a practical solution for the wide-beam antenna design with potential applications in wide-area wireless communication systems.

In contribution 13, Cai and Tong introduce a wideband circularly polarised cross-fed magneto-electric dipole antenna. The simple geometry utilises open slots between cross-fed microstrip patches to achieve circular polarisation and high stable gain across a wide frequency band. The proposed antenna, with a wide impedance bandwidth and in-band 3-dB axial ratio bandwidth, represents potential applications in wireless communication systems.

The paper by Srikar et al. (contribution 14) presents a cognitive radio-integrated antenna system with 1 sensing and 24 communication antennas. The system, catering to different operating bands, demonstrates good diversity characteristics and mutual coupling. The proposed design offers a comprehensive solution for spectrum utilisation efficiency in cognitive radio applications.

The work by Ayaz et al. (contribution 15) introduces a conformal cylindrical phased array antenna excited with composite right-/left-handed (CRLH) phase shifters. The novel aspect involves embedding magneto-static field-responsive micron-sized particles into the CRLH phase shifter structure, enabling variable phase shifts without increasing the insertion loss or phase error. The proposed antenna, operating in the C-band (5–6 GHz), exhibits low insertion loss and phase error, making it suitable for a printed and flexible electronics design. The prototype of the cylindrical phased array, with the particle-embedded CRLH phase shifters, demonstrates a close agreement between simulated and measured results, presenting a promising solution for conformal array applications.

The research by Nurhayati et al. (contribution 16) delves into the design of a 1 × 2 MIMO Palm Tree Coplanar Vivaldi Antenna in the E-Plane, aiming to overcome challenges related to mutual coupling and grating lobes. The authors employ diverse patch structures, such as Back Cut Palm Tree (BCPT) and Horizontal Wave Structure Palm Tree (HWSPT), demonstrating superior return loss and mutual scattering. Additionally, their incorporation of Metamaterial Lens Palm Tree (MLPT) into radar applications further extends the antenna’s utility.

Bohao Tang et al. (Contribution 17) present an Evolutionary Computation approach for the Sparse Synthesis Optimization of Concentric Circular Antenna Arrays (CCAAs). By introducing hybrid solution initialisation and crossover methods, the proposed algorithm optimises CCAAs to reduce sidelobes while turning off specific antennas, addressing challenges related to overhead and excessive sidelobes in these arrays.

In contribution 18, by Ning Zhang et al., a dual-polarisation dipole antenna for a cylindrical phased array in Ku-Band is introduced. The dual-layer structured antenna, composed of butterfly shaped dipoles, demonstrates improved isolation between ports and effective scanning capabilities. The proposed design, applied in a 32-element cylinder array, indicates its potential for conformal devices in Ku-band frequencies.

Marcellin Atemkeng et al. (contribution 19) focus on the expansion of the African Very Long Baseline Interferometry (AVN) Network, particularly in the central African region. Analysing the scientific impact of additional antennas in countries like Cameroon and Chad, the paper emphasises the economic and human capital impacts of radio interferometers, contributing to the broader success of the AVN project.

In contribution 20, by Madiha Farasat et al., a simple yet effective approach for scattering suppression in multiband base station antennas is presented. By introducing a novel horizontal and vertical radiating element, the authors successfully mitigate high band pattern distortions, providing improved return loss and comparable pattern performance over the entire frequency band.

Irfan Ullah et al. (contribution 21) explore adaptive beamforming patterns of microstrip patch antenna arrays on flexible surfaces, emphasising the importance of conformal and self-adapting beamforming in the era of wireless spectrum growth. Their work offers insights into efficient and robust conformal phased-array antennas with multiple beamforming capabilities.

Chunli Wang et al. (contribution 22) propose novel coplanar meta-surface-based substrate-integrated waveguide antennas for K-Band beam scanning. Their innovative designs leverage coplanar rhombus- and hexagon-shaped meta-surfaces, providing low reflection and wide bandwidth, thus paving the way for highly directive scanning radiation in mm-Wave applications.

In contribution 23, by Aiting Wu et al., a compact four-port MIMO antenna for UWB applications is introduced. Utilising a polarisation diversity approach, the authors achieve a compact design with small dimensions and demonstrate promising performance in terms of impedance bandwidth, isolation, and gain, making it suitable for UWB applications.

Eunice Oluwabunmi Owoola et al. (contribution 24) propose an advanced marine predator algorithm (AMPA) to optimise non-uniform CAA beam patterns. The algorithm effectively optimises amplitude current and inter-element spacing for CAAs with varying element counts, achieving superior peak sidelobe level (SLL) suppression and convergence rates compared to other algorithms.

In contribution 25, by Bancha Luadang et al., a portable Yagi–Uda-based directional antenna for digital terrestrial television (DTT) is proposed. Simulations and tests show an impedance bandwidth of 75.93%, gain from 2.69 to 4.84 dBi, and a unidirectional radiation pattern. The radome has minimal impact, and outdoor/indoor tests yield power measurements at 514 MHz of 38.4 dBµV (−70.4 dBm) and 26.6 dBµV (−82.2 dBm), with carrier-to-noise ratios (C/N) of 11.6 dB and 10.9 dB. 

Boasting a compact size of 30 × 18 × 1.6 mm^3^, the ultra-wideband (UWB) multiple-input, multiple-output (MIMO) antenna by Weidong Mu et al. (contribution 26) exhibits flower-shaped radiating components, providing high isolation and excellent performance across the entire operation band of 4.3–15.63 GHz. The proposed design holds promise for diverse UWB applications.

In contribution 27, by Abdul Wajid et al., a dual-band wearable patch antenna with split-ring resonator (SRR)- and electromagnetic bandgap (EBG)-based designs is presented. The SRR-based antenna demonstrates improved gain, surface wave suppression, and compactness, showcasing its potential for wearable sensor networks and IoT applications.

Contribution 28 by Xianjin Yi et al. introduces a dual-band high-gain shared-aperture antenna that integrates Fabry–Perot and reflect-array mechanisms. Operating in both the S-band and X-band, the antenna achieves impressive gains with good isolation between the two frequency bands, making it an attractive candidate for high-performance communication systems.

The paper by Rozenn Allanic et al. (contribution 29) introduces polarisation-reconfigurable patch antennas using semiconductor-distributed doped areas (ScDDAs). The co-design method presented enables the optimisation of both the antenna and the ScDDAs, offering a practical and efficient solution for polarisation reconfiguration.

Contribution 30 by Yuefei Yan et al. addresses the growing importance of electromechanical coupling in high-frequency communication base station antennas. The authors establish a comprehensive channel capacity model, considering factors like positional shift, attitude deflection, and temperature change, providing insights crucial for the design and manufacture of advanced communication systems.

The research by Fengan Li et al. (contribution 31) introduces a novel metasurface comprising complementary units, enabling multi-band dual polarisation conversion. The design not only achieves remarkable frequency bands for linear and linear-to-circular polarisation conversion, but also shows radar cross-section (RCS) reduction capabilities, extending its application to multiple microwave frequency bands.

In the realm of 5G communication systems, Syed Aftab Naqvi and team (contribution 32) present a dual-band metamaterial-based absorber operating at 24 GHz and 28 GHz. Addressing the challenges of massive MIMO techniques, their absorber design enhances gain and spatial multiplexing while effectively isolating adjacent antennas. This innovative absorber holds promise for compact 5G devices by preventing unwanted interactions between antennas.

Kerlos Atia Abdalmalak and colleagues (contribution 33) contribute a unique feeding method for linear dielectric resonator antenna (DRA) arrays. By utilising standing waves and discrete metallic patches, the authors achieve a high-gain DRA array with low losses, presenting a cost-effective and compact design. The proposed 3D-printed structure demonstrates high efficiency, making it a noteworthy advancement in feeding techniques.

In contribution 34, Zhiyi Li et al. present a low-profile wideband magnetoelectric (ME) dipole antenna. The design involves the intricate bending of structures to achieve a reduced antenna height, while maintaining wideband properties. The relative bandwidth for VSWR < 2.0 and low boresight gain drop make this antenna suitable for applications requiring limited height and wideband characteristics.

Chao Ni et al. (contribution 35) propose a filtering slot antenna using characteristic mode analysis (CMA). Their approach involves analysing and designing characteristic magnetic currents to achieve a wide filtering bandwidth with stable gain. The fabricated prototype validates the design process, showing promising results for applications where stable gain and wide filtering are crucial.

Niamat Hussain et al. (contribution 36) contribute a conformal frequency-reconfigurable antenna catering to smart portable devices. With a focus on flexibility and multi-frequency operation, the antenna employs a coplanar waveguide-fed slotted circular patch. The integration of a frequency-reconfigurable element enhances its adaptability to various operating bands, making it suitable for modern wireless devices.

Addressing the demands of 5G millimetre-wave communications, Hussain Askari and colleagues (Contribution 37) present a wideband, circularly polarised magnetoelectric (CP ME) dipole antenna operating at 28 GHz. The unique geometry, including metallic plates and hook-shaped strips, enables stable gain and wideband characteristics. The antenna’s compact footprint makes it well-suited for 5G smart devices and sensors.

Shenko Chura Aredo et al. (contribution 38) tackle the challenges associated with massive MIMO systems, proposing hardware-efficient solutions with optimal antenna selection. By evaluating low-resolution digital-to-analogue conversion and antenna selection techniques, the authors aim to reduce power consumption and enhance energy efficiency in massive MIMO systems.

The study by Ruisi Ge et al. (contribution 39) explores the influence of conformal metasurfaces on passive beam steering. The research introduces a passive approach to beam steering, utilising conformal metasurfaces on conventional patch antennas. The simplicity of the proposed system, combined with its passive nature, holds promise for low-power consumption beam steering systems.

The research from Faxiao Sun et al. (contribution 40) introduces a novel rotating shutter antenna designed for ultra-low-frequency (ULF) communication, with a focus on its potential application as a transmitter for magnetic induction (MI) underground communication systems. The authors employ advanced simulations and experiments to validate the antenna’s performance, showcasing its ability to generate 2FSK signals in the ULF band. The proposed rotating shutter antenna holds promise for enhancing communication in challenging environments, such as underground spaces.

The article by Kazuhiro Honda (contribution 41) delves into the over-the-air testing of a massive multiple-input multiple-output (MIMO) antenna. The paper presents an innovative testing method involving a full-rank channel matrix created through a fading emulator with a minimal number of scatterers. By virtually positioning scatterers through antenna rotation, the study demonstrates a practical approach to assessing the performance of massive MIMO systems. This methodology offers insights into the real-world functionality of large-scale antenna arrays.

Marek Garbaruk et al. (contribution 42) propose a planar four-element ultrawideband (UWB) antenna array designed for the 6–8.5 GHz UWB frequency band. The symmetrical structure and elliptical-shaped radiators, fed by a stripline excitation network, contribute to uniform power distribution. With measured gains ranging from 6.4 to 10.8 dBi, the UWB antenna array exhibits favourable impedance matching. This research addresses the demand for high-performance antennas in the European Commission’s designated UWB frequency band.

Shimaa A. M. Soliman et al. (contribution 43) offer insights into the analysis and design of an X-band reflect-array antenna tailored for a medium Earth orbit (MEO) remote sensing satellite system. The study explores various reflect-array configurations, including broadside and tilted pencil beam options, optimising the antenna for a nearly constant response across the coverage area. The use of a Yagi–Uda array and a genetic algorithm (GA) optimisation method demonstrates an efficient design process for achieving a flat-top radiation pattern.

In contribution 44, Raffaele Moretta et al. address the challenge of efficiently sampling the field radiated by a circumference arc source. The paper introduces a methodology to determine the minimum number of basis functions required for accurate representation and proposes an interpolation formula that optimally exploits non-redundant field samples. This work contributes to advancing the efficiency of field measurement techniques, crucial for applications where acquisition time is a limiting factor.

Mingcong Xie et al. (contribution 45) tackle the complex problem of aperture-level simultaneous transmit and receive (ALSTAR) with a digital phased array. The authors propose an adaptive random group quantum brainstorming optimisation (ARGQBSO) algorithm to simplify the array design, addressing the isolation between transmit and receive apertures. This innovative algorithm demonstrates robust performance, reducing complexity and enhancing overall efficiency in ALSTAR systems.

Hamdi Bilel and Aguili Taoufik (contribution 46) present a novel formulation based on Floquet spectral analysis for the almost-periodic modulation of massive finite and infinite strongly coupled arrays. The study has significant implications for applications such as dense-massive-MIMO, intelligent-surfaces, and future wireless technologies (5G and 6G). The numerical methods adopted, including the method of moments, pave the way for the advanced modelling of antenna structures in small areas with a large number of elements.

Collectively, these contributions demonstrate the breadth and depth of contemporary antenna research, addressing challenges and pushing the boundaries of what is possible in diverse applications. The innovative methodologies and findings presented in this Topic contribute to the ongoing evolution of antenna technologies, playing a vital role in shaping the future of wireless communication and sensing systems.

## 3. Conclusions

The odyssey through contributions 1 to 46 in this Topic has not only illuminated the cutting edge of antenna research but has also unfurled a vibrant tapestry of groundbreaking discoveries that significantly redefine the boundaries of what was once deemed possible in the realms of wireless communication, sensing systems, and beyond. Each contribution stands as a testament to the collective ingenuity and tireless efforts of researchers dedicated to pushing the frontiers of knowledge. The diverse array of antennas, from the non-linear chaotic optimisation algorithm to the innovative Floquet spectral analysis, shows the versatility and adaptability of antenna technologies. This collection encapsulates the dynamic and ever-evolving nature of the field, demonstrating how research endeavours continue to reshape our understanding and utilisation of antennas in various applications. As we traverse this intellectual landscape, it becomes evident that the relentless pursuit of excellence is not merely a shared ethos but a driving force propelling the evolution of wireless technologies into uncharted territories. In essence, the odyssey through these contributions serves as a compass, guiding us toward a future where antennas will play an even more pivotal role in shaping the landscape of global connectivity, sensing, and communication.

As we navigate the frontiers of antenna advancements, the prospect of future research beckons with enticing challenges and unexplored possibilities. One promising avenue lies in further enhancing the adaptability and robustness of antennas for emerging applications such as the Internet of Things (IoT) and smart cities. Exploring sustainable materials and fabrication techniques to reduce environmental impact is another crucial direction. Additionally, the integration of artificial intelligence and machine learning algorithms for the autonomous optimisation of antenna parameters holds immense potential [9]. The advent of 6G technologies and beyond will likely demand antennas that can seamlessly operate across a broader range of frequencies, with increased energy efficiency and reduced interference. The quest for antennas capable of supporting massive MIMO systems and intelligent surfaces represents a frontier in which interdisciplinary collaboration and innovation will play a pivotal role. In this ever-evolving field, embracing the challenges of real-world deployment scenarios, including the influence of environmental factors and dynamic interference, will be essential for pushing the boundaries of what can be achieved with antenna technologies [10]. As we stand at the precipice of a new era in wireless communication, the contributions within this collection not only mark significant milestones but also lay down the gauntlet for the exciting journey that lies ahead.

We wish to express our sincere gratitude to the esteemed authors whose remarkable contributions have profoundly enriched this MDPI Topic. Their invaluable research has not only propelled the field of antennas forward significantly, but has also contributed to the broader landscape of knowledge in impactful ways. Our deepest thanks extend to the diligent reviewers whose insightful comments and constructive feedback have played a pivotal role in elevating the quality of the articles presented here. Their expertise and unwavering dedication have been instrumental in ensuring the robustness and precision of the published works.

Acknowledgment goes to the dedicated editorial board and the supportive editorial offices of MDPI’s *Electronics*, *Future Internet*, *Sensors*, and *Telecom* journals. Their consistent guidance and tireless assistance throughout the entire publication process have been indispensable, contributing significantly to the realisation of this comprehensive collection. We are confident that readers will find these papers not only informative but also enlightening, providing valuable insights into the dynamic and evolving realm of antennas. Looking ahead, we eagerly anticipate continued collaboration and future contributions from researchers in the field. Together, we aim to further advance and explore new frontiers in antenna technology. To all involved, we extend our heartfelt appreciation for your remarkable efforts and unwavering dedication to the advancement of knowledge in this field.
